# *Trichoderma* spp. Associated with Teosinte (*Zea mays* spp. *mexicana*) Rhizosphere Exhibit Potential Plant Growth-Promoting and Antagonistic Functional Traits

**DOI:** 10.3390/jof12060392

**Published:** 2026-05-29

**Authors:** Luis Angel Morales-Mora, Ignacio Eduardo Maldonado-Mendoza, Soley Berenice Nava-Galicia, Omar Romero-Arenas, Analilia Arroyo-Becerra, Miguel Angel Villalobos-López, Diana Verónica Cortés-Espinosa, Martha D. Bibbins-Martínez

**Affiliations:** 1 Centro de Investigación en Biotecnología Aplicada, Instituto Politécnico Nacional, Carretera Estatal Sta, Inés Tecuexcomac-Tepetitla, Km 1.5, Tepetitla de Lardizábal 90700, Tlaxcala, Mexico; lmoralesm2300@alumno.ipn.mx (L.A.M.-M.); snava@ipn.mx (S.B.N.-G.); alarroyo@ipn.mx (A.A.-B.); mvillalobosl@ipn.mx (M.A.V.-L.); dcortes@ipn.mx (D.V.C.-E.); 2 CIIDIR-Unidad Sinaloa, Departamento de Biotecnología Agrícola, Instituto Politécnico Nacional, Boulevard Juan de Dios Bátiz Paredes, No. 250, Guasave 81101, Sinaloa, Mexico; 3 Centro de Agroecología, Instituto de Ciencias, Benemérita Universidad Autónoma de Puebla, Edificio VAL 1, Km 1.7 Carretera a San Baltazar Tetela, San Pedro Zacachimalpa 72960, Puebla, Mexico; biol.ora@hotmail.com

**Keywords:** *Trichoderma*, teosinte, endophytes, maize, plant growth promotion, biocontrol

## Abstract

Wild maize (teosinte) has been reported to be less susceptible to biotic and abiotic stresses than its modern relative, corn. The composition of the teosinte root microbiome may be linked to traits such as drought tolerance and pest resistance. *Trichoderma* spp. are ubiquitous saprotrophic fungi found in the plant rhizosphere, enhancing host plant growth and crop productivity while alleviating biotic and abiotic stresses. The present study identified ten *Trichoderma* fungal isolates associated with the rhizosphere microbiome of teosinte (*Zea mays* spp. *mexicana*) and performed in vitro screening to assess both their multi-trait plant growth-promoting activities and their biological control potential against the phytopathogens *Aspergillus flavus* and *Fusarium verticillioides*. Additionally, interaction tests were conducted to evaluate the phytostimulant effect of *Trichoderma* spp. on maize (*Zea mays*) seed germination. Taxonomic and phylogenetic analysis identified five different *Trichoderma* species: *T. rifaii* (TA and TH); *T. azevedoi* (TB and TI); *T. afroharzianum* (TE); *T. hamatum* (TF and TG); and *Trichoderma* sp. (aff. *bannaense*) (TC, TD, and TJ). Partial least squares discriminant analysis revealed the isolates TF, TG, and TJ to have the highest potential for use as biocontrol and biostimulant agents. The present study is the first to examine *Trichoderma* species associated with the teosinte microbiome, and the results suggest that *Trichoderma* isolates are a potential sustainable alternative for improving maize cultivation.

## 1. Introduction

The rhizosphere of teosinte (*Zea mays* spp. *mexicana*), a wild relative of modern maize, has emerged as a useful model for studying functional microbial diversity, and its potential applications in sustainable agriculture are also attracting increasing research interest [1]. Unlike domesticated maize, teosinte has not undergone intensive anthropogenic selection pressures, enabling it to retain greater genetic and microbiological diversity, thus positioning it as an ancestral reservoir of beneficial microorganisms that play key roles in plant growth and protection [2]. Recent studies employing metagenomic techniques, high-throughput sequencing, and microbial isolation have revealed that both the teosinte rhizosphere and endosphere harbor bacterial and fungal communities that are significantly different from and often more complex than those found in modern maize cultivars [3]. Endophytic genera such as *Pseudomonas*, *Bacillus*, and *Enterobacter* have been characterized in teosinte, contributing to nutrient solubilization, siderophore and phytohormone production, and pathogen suppression [1,3,4,5,6,7]. In addition, endophytic fungi isolated from teosinte, including species of *Aspergillus*, *Gliocladium*, and *Rhizopus*, have been reported to enhance nutrient uptake efficiency and promote plant growth under reduced fertilizer conditions [8]. However, there are no direct reports of *Trichoderma* spp. isolated from the teosinte rhizosphere; existing evidence suggests that this genus may be present in the latter, given the microbial richness associated with this wild host [1]. In this context, bioprospecting Teosinte-associated strains (TAS) *Trichoderma* sp., from the teosinte rhizosphere is a compelling strategy as these fungi are widely recognized for their versatility, which includes use as biocontrol agents (BCAs), bio-stimulants, and plant growth-promoting fungi (PGPF) [9,10,11,12,13]. *Trichoderma* spp. are cosmopolitan fungi capable of establishing mutualistic relationships with diverse hosts, rapidly colonizing the rhizosphere, solubilizing nutrients, inducing systemic defense mechanisms, suppressing phytopathogens, and enhancing the physical and functional structure of the soil, ultimately leading to sustained increases in agricultural productivity [14,15,16]. These multifunctional traits make *Trichoderma* a promising tool for modern sustainable agricultural frameworks that aim to reduce the indiscriminate use of agrochemicals, which negatively affect both the environment and human health [17]. Isolating *Trichoderma* TAS from wild sources, such as teosinte, offers an innovative pathway to strengthen agroecosystem resilience and restore ecosystem functions lost during maize domestication [18]. In contrast, the widespread use of any *Trichoderma* strains has led to adverse effects on soil health, including the displacement of indigenous microorganisms [19,20,21]. Therefore, we hypothesize that *Trichoderma* strains associated with the teosinte rhizosphere (TAS) exhibit superior multifunctional activities as a result of co-adaptation with a stress-resilient wild host. Accordingly, the objectives of this study were to isolate and identify teosinte-associated *Trichoderma* strains to evaluate their in vitro plant growth-promoting and antagonistic traits and assess their effects on maize seedling development.

## 2. Materials and Methods

### 2.1. Sample Collection and Isolation of Trichoderma

Rhizosphere samples were collected from wild *Zea mays* spp. *mexicana* plants growing in non-agricultural, agrochemical-free soils in San Luis Coyotzingo, Huejotzingo, Puebla. Sampling was conducted at three georeferenced sites—Zone 1 (19°12′06.4″ N, 98°24′49.3″ W), Zone 2 (19°11′22.3″ N, 98°25′26.9″ W), and Zone 3 (19°11′00.7″ N, 98°25′47.6″ W)—during two seasonal periods (February and September) in 2023. Rhizospheric soil sampling was conducted following the protocol described in [22]. At each site, ten random subsamples were collected from the root zone and homogenized to obtain composite samples of 200 g in triplicate. Additionally, root samples from five plants per site were collected, placed in labelled bags, and transported at 4 °C to CIBA-IPN Tlaxcala for subsequent analysis.

Rhizospheric TAS of *Trichoderma* spp. were obtained from surface-sterilized secondary roots using *Trichoderma* selective medium (TSM) [23] and potato dextrose agar (PDA) supplemented with 0.5% Triton X-100, following the protocols described by Andrade-Hoyos et al. [24] and Steyaert et al. [25]. Colonies exhibiting typical *Trichoderma* morphology were purified by applying the hyphal tip technique on PDA. Reference non-Teosinte associated strains (non-TAS) comprised *T. harzianum* TH3, *T. asperellum* TASP, *T. atroviride* IMI 206040 TWT, and *T. koningiopsis* TK11. These isolates, obtained from the roots of *Persea americana* (avocado), were previously characterized as biocontrol agents against *Fusarium oxysporum*, *F. solani*, *Pestalotiopsis* sp., and *Phytophthora cinnamomi* [24].

### 2.2. Identification of Trichoderma Isolates

#### 2.2.1. Morphological Characterization

The morphological characterization performed in the present study was conducted on potato dextrose agar (PDA; 200 g potato infusion, 20 g glucose, 15 g agar, 1 L distilled water), corn meal dextrose agar (CMD; 40 g cornmeal, 20 g glucose, 18 g agar, 1 L distilled water), and synthetic low-nutrient agar (SNA; 1 g KH_2_PO_4_, 1 g KNO_3_, 0.5 g MgSO_4_·7H_2_O, 0.5 g KCl, 0.2 g glucose, 0.2 g sucrose, 18 g agar, 1 L distilled water). The fungal isolates were characterized following the protocol described in [26], including incubation at 25 °C for 72 h under dark conditions, after which colony radial growth, texture, pigmentation, sporulation pattern, and the presence of spores or chlamydospores were recorded. Microscopic characterization was conducted using SNA microcultures incubated for 48–72 h, fixed with 10% potassium hydroxide, and stained with malachite green. Phialide, conidia, and chlamydospore morphology were described according to [27,28], using optical microscopy (Olympus BX50, Olympus Corporation, Tokyo, Japan) and digital measurements obtained via Fiji-ImageJ software package (Java 2 v1.5).

#### 2.2.2. Molecular Identification

Genomic DNA was extracted from mycelium grown on PDA for five days at 27 °C in the dark, following the protocols described by [29,30]. The extraction used TES buffer, 20% CTAB, and β-mercaptoethanol, followed by chloroform: isoamyl alcohol purification and precipitation with ammonium acetate and isopropanol. The DNA recovered was washed with 70% ethanol, dried, and resuspended in diethyl pyrocarbonate (DEPC)-treated water. DNA quality and concentration were assessed by agarose gel electrophoresis and spectrophotometry (Nanodrop 1000 spectrophotometer, Thermo Fisher Scientific, Wilmington, DE, USA). Partial sequences of three DNA barcoding loci (ITS, TEF1, and RPB2) were amplified via polymerase chain reaction (PCR) using a Bio-Rad T100 thermal cycler. The primer pairs F-ITS5 (GGAAGTAAAAGTCGTAACAAGG)-R-ITS4 (TCCTCCGCTTATTGATATGC), F-EF1 (ATGGGTAAGGARGACAAGAC)-R-EF2 (GGARGTACCAGTSATCATGTT), and F-RPB2-5f (GAYGAYMGWGATCAYTTYGG)-R-RPB2-7cr (CCCATRGCTTGTYYRCCCAT) were used. PCR amplifications consisted of an initial denaturation at 95 °C for 3 min, followed by 35 cycles of denaturation at 95 °C for 15 s, annealing at 53 °C for 15 s, and extension at 72 °C for 1 min, with a final extension at 72 °C for 5 min, as described in [31]. The PCR products were visualized on 1% agarose gels stained with Midori Green Advance, with DNA concentration adjusted to 50 ng/mL. Samples were submitted for bidirectional Sanger sequencing at the National Laboratory for Agricultural, Medical, and Environmental Biotechnology (LANBAMA), San Luis Potosí, Mexico.

#### 2.2.3. Phylogenetic Analyses

The *Trichoderma* isolates were subjected to phylogenetic analysis conducted following the methodology described by [32], with specific adjustments applied for this fungal group. DNA sequences from both strands were assembled using BioEdit v7.0.5 [33] and compared against GenBank entries via the BLASTn+2.17.0 algorithm [34]. A total of 34 representative *Trichoderma* sequences were retrieved, aligned with Muscle [35] in MEGA v7.0.26 [36], and trimmed at both ends to standardize sequence length. Sequences from the ITS, TEF1-α, and RPB2 loci were concatenated using Mesquite v2.0 and analyzed via Bayesian inference in MrBayes v3.2.6 [37], with four MCMC chains running for 1,000,000 generations, sampling every 1000th cycle and discarding the initial 25% as burn-in. The following evolutionary models were selected in MEGA v12 [38] based on the Akaike Information Criterion (AIC): K2+G+I for ITS; TN93+G+I for TEF1-α; and T92+G+I for RPB2. The resulting phylogenetic tree, derived from the concatenated matrix, was visualized in FigTree v1.4.4 [39], using *Pestalotiopsis hollandica* as the outgroup (GenBank: MH855436.1, MH554936.1, KM199481.1). A shared evolutionary history among loci was assumed, and MCMC convergence was verified using partition standard deviation. Models were applied to each partition, and nodes with posterior probabilities ≥ 95% were considered well supported. The sequences generated by the present study were then deposited in the National Center for Biotechnology Information (NCBI) GenBank (https://www.ncbi.nlm.nih.gov), and the accession numbers are provided in Table 1.

### 2.3. In Vitro Evaluation of Plant Growth Promotion Activities

#### 2.3.1. Phosphate Solubilization Assay

The phosphate-solubilizing capacity of the isolates was evaluated using qualitative and quantitative approaches in Pikovskaya media (PVK; 10 glucose, 0.5 yeast extract, 0.5(NH_4_)_2_SO_4_, 0.1MgSO_4_·7H_2_O, 5Ca_3_(PO_4_)_2_, 0.2KCl, 0.002MnSO_4_·2H_2_O, 0.002FeSO_4_·7H_2_O, 15 agar, 1 L distilled water) [40]. The qualitative analysis used PVK agar plates prepared according to the method described in [41]. The soluble inorganic phosphate (IPS) level was quantified using a protocol adapted from [42]. The culture was established in 250 mL Erlenmeyer flasks containing 100 mL PVK broth (pH 7.0), with each flask inoculated with four mycelial pellets (4 mm in diameter) obtained with a steel punch from the periphery of *Trichoderma* spp. cultures grown on PDA for seven days at 28 °C. All flasks were incubated at 28 °C for four days under constant agitation. Following incubation, the cultures were centrifuged at 8000× *g* for 20 min, and the concentration of available phosphorus was then determined using the molybdenum blue method [43]. Phosphate solubilization was quantified using a standard curve generated with potassium phosphate dibasic (K_2_HPO_4_) (y = 0.0166x + 0.0124; R^2^ = 0.98), with results expressed as phosphate equivalents (μg/mL). Non-inoculated sterile PVK broth containing insoluble tricalcium phosphate (Ca_3_(PO_4_)_2_) was included as a negative control, which yielded no detectable soluble phosphate. At the end of the assay, the culture pH was recorded using a calibrated pH meter.

#### 2.3.2. Organic Acid Production

Organic acid production was evaluated using Pikovskaya media [40] supplemented with bromocresol purple (PVK + BP), a pH-sensitive indicator [43]. Plates were inoculated with a mycelial pellet (4 mm in diameter) obtained with a steel punch from the periphery of *Trichoderma* spp. cultures grown on PDA for three days at 28 °C and incubated at 28 °C. Images of the yellow halos formed 24 h after fungal inoculation, indicative of medium acidification [44], were recorded and used to calculate the organic acid production index (OAPI) on the plate. For this purpose, the average values for each halo radius were determined, and the color hue was measured using FIJI-ImageJ software [45] and the H channel of the HSV color model. As a chromatic reference, a pH gradient ranging from 5 to 7 was prepared using bromocresol purple. The OAPI calculation was performed using the following formula:

(1) OAPI = r (mm) × H/100

where r corresponds to the radial yellow zone and H is the chromatic color value of the yellow zone.

#### 2.3.3. Screening for Siderophore Production

Siderophore production was assessed using the Chrome Azurol S (CAS) (Sigma-Aldrich, Merck KGaA, Darmstadt, Germany) colorimetric assay [46] in accordance with [47] and using MM9 as the basal media. Three mycelial discs, obtained from three-day-old isolates, were inoculated per plate to monitor halo formation or color shifts from blue to orange, yellow, purple, dark violet, or red [48]. A non-inoculated plate served as the control. The halo radius was recorded to calculate the siderophore production index (SPI), as adapted from that set out in [49]:

SPI = r (mm) + rC (mm)/rC(mm)

where r corresponds to the siderophore halo radius and rC to the radial growth of the fungal colony.

#### 2.3.4. Screening for Indole Acetic Acid Production

The production of indole-3-acetic acid (IAA) by the *Trichoderma* isolates was determined using a colorimetric technique based on the Salkowski method, following the modified protocol reported by [50]. Briefly, the fungal isolates were cultured in PDB with and without 0.5 mg/mL L-tryptophan supplementation, with each 50 mL culture obtained, inoculated with 500 µL of a spore suspension (10^6^ conidia/mL), and incubated for 4 days at 28 ± 2 °C under constant agitation at 120 rpm. After incubation, the medium was filtered (Whatman No. 4, Sigma-Aldrich, Merck KGaA, Darmstadt, Germany) and centrifuged at 12,000 rpm for ten minutes to obtain the supernatant, which was mixed with Salkowski reagent (150 mL concentrated H_2_SO_4_, 250 mL distilled water, and 7.5 mL of 0.5 M FeCl_3_·6H_2_O) at a 1:2 (*v*/*v*) ratio and incubated in the dark at room temperature for 30 min. Absorbance was measured at 530 nm using a UV–vis spectrophotometer (Thermo Scientific Genesys 10S, Thermo Fisher Scientific, Madison, WI, USA), while IAA concentration was estimated using a standard curve (10–100 µg/mL), as described by [51]. Uninoculated PDB media treated with the same reagent served as the blank control.

#### 2.3.5. Impact of *Trichoderma* spp. on Maize Seedling Growth Measured via Paper Towel Assay

To evaluate the growth-promoting effect of various *Trichoderma* isolates, maize (criollo) seeds collected in Teolocholco, Tlaxcala, Mexico, were used. Before sowing, seeds were surface-sterilized according to the protocol described by [52,53] and then immersed for 2 h in conidial suspensions prepared at a concentration of 1 × 10^6^ conidia/mL, supplemented with 1.5% carboxymethylcellulose to improve inoculant adhesion [54,55]. After treatment, the seeds were dried under controlled conditions (at 25 °C on sterile paper towels, maintained under aseptic conditions inside a laminar flow cabinet for at least 1 h). The experiment was conducted under a completely randomized design comprising 15 treatments (T) with 3 replicates, with each replicate consisting of 21 seeds. Treatments TA–TJ corresponded to seeds inoculated with *Trichoderma* TAS, while TH3, TK11, TWt, and TASP corresponded to seeds inoculated with *Trichoderma* non-TAS. The control treatment (CT) consisted of non-inoculated seeds subjected to the same surface-sterilization and immersion procedures using a sterile carrier solution without conidia.

The seeds were sown under controlled conditions using a modified version of the rolled paper towel method described by [53]. Germination paper, previously moistened with 0.5% commercial sodium hypochlorite solution, was administered in double layers on which the seeds were placed and then covered with another sheet, forming a type of ‘sandwich’ arrangement. Approximately 7 seeds were placed per sheet, each of which was rolled firmly, placed vertically in a beaker containing 100 mL of sterile water, covered with another inverted beaker, sealed with Parafilm, and fitted with a cotton-gauze plug to allow gas exchange. The effectiveness of the growth promotion was assessed on day 7 of the experiment by measuring key physiological parameters, including shoot length, primary and secondary root length, and dry biomass accumulation.

### 2.4. In Vitro Screening of Antagonistic Activity of Trichoderma *spp.*

#### 2.4.1. Antagonism Assays in Dual Culture

The antagonistic effects of *Trichoderma* spp. against *A. flavus* PR96B [56] and *F. verticillioides* PO3 [57] were evaluated using dual-culture (DC) assays, following the methodology described by [58]. A 90 mm Petri dish containing PDA medium was inoculated with mycelial plugs (obtained from a three-day-old colony of the phytopathogen of interest) placed 7 cm apart. The controls comprised phytopathogens grown separately on dishes without the antagonist. The growth and inhibition characteristics were monitored every 24 h, and images were taken at each time point to assess the interaction. The images were analyzed using the FIJI-ImageJ software package (Java 2 v1.5) [45], with data obtained from the calculation of the surface area of the fungi and then used to calculate the mycelial growth inhibition percentage via the following formula: IP = (A1 − A2/A1 ×100%), where A1 is the surface area of the control phytopathogen and A2 is the surface area of the pathogen throughout the antagonism process.

#### 2.4.2. Antagonism Assays by Volatile Compounds (VOCs)

The impact of the volatile organic compounds (VOCs) produced by *Trichoderma* spp. on phytopathogen growth was evaluated using two-compartment Petri dishes, following the protocol described by [25]. Each compartment contained 5 mL of PDA medium, with one side inoculated with the phytopathogen (5 µL of a spore suspension at 1 × 10^6^ spores/mL) and the other with a 5 mm diameter mycelial plug of *Trichoderma* spp. cultured for 3 days. The plates were sealed with Parafilm and incubated at 27 °C for 96 h under a 12 h light/12 h dark photoperiod. The surface area of the phytopathogen growth was measured in both the presence and absence of *Trichoderma* VOCs, and the inhibition percentage was calculated according to the following formula: IP = (A1 − A2/A1 ×100%), where A1 is the surface area of the control phytopathogen and A2 is the surface area of the pathogen throughout the VOC assay. Each treatment was performed in triplicate.

#### 2.4.3. Antagonism Assays by Nonvolatile Compound Assays

The production of diffusible compounds by *Trichoderma* spp. in response to the phytopathogens of interest was evaluated using the antibiosis assay described by [25]. Petri dishes containing PDA media were covered with cellophane, centrally inoculated with fresh *Trichoderma* mycelial plugs (5 mm), and then incubated at 27 °C for 24–30 h. After the mycelium and cellophane had been removed, the phytopathogen was inoculated onto the previously exposed medium. The control consisted of phytopathogen inoculation on PDA medium without prior contact with *Trichoderma*. Pathogen growth was monitored for 72 h, and antibiosis-mediated inhibition was calculated using FIJI-ImageJ [45], as described in the dual-culture assay.

### 2.5. Data Analysis

To evaluate the functional performance of the 14 *Trichoderma* strains, dependent variables were assessed in three categories: biocontrol activity (DC, antibiosis, and VOCs); plant growth promotion (IPS, OAPI, SPI, and IAA); and seed germination parameters (shoot length, primary and secondary root length, and shoot/root biomass). A one-way ANOVA was applied for each variable, followed by Duncan and Tukey’s HSD tests for multiple comparisons (*p* ≤ 0.05). Strain variability and clustering were further analyzed using partial least squares discriminant analysis (PLS-DA) on Z-score-normalized data, with model robustness assessed by cross-validation and permutation tests (*n* = 100). A hierarchical heatmap (Z-score-normalized) was generated to visualize the multivariate distribution of biocontrol, plant growth-promotion, and seedling development traits across isolates using Ward’s method and Euclidean distance. All analyses were performed in IBM SPSS Statistics v.27 [59] and MetaboAnalyst 5.0 [60]. Additionally, to characterize unsupervised patterns of functional differentiation among strains, principal component analysis (PCA) was performed using auto-scaled data. PCA score matrices were subsequently subjected to *k*-means clustering to identify emergent groups based on Euclidean distances. These unsupervised analyses complemented the supervised PLS-DA by providing an independent assessment of multivariate structure and natural group separation among isolates.

## 3. Results

### 3.1. Isolation and Morphological Identification of Trichoderma Strains

**Figure 1:**
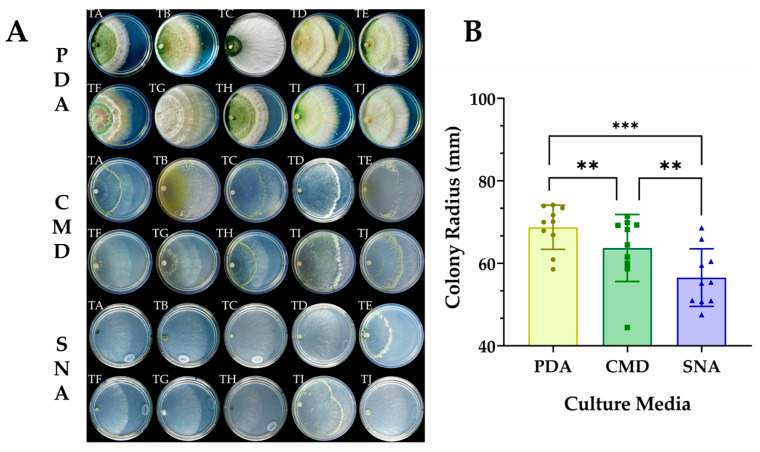
Mycelial growth of *Trichoderma* spp. (**A**) Colony morphology on PDA, CMD, and SNA media after 72 h incubation at 27 °C. (**B**) Comparison of colony radius (mm) among isolates after 72 h incubation on PDA, CMD, and SNA. Bars represent the mean ± standard deviation (SD). The asterisks (*** and **) represent high and moderate significant differences, respectively, among all the means obtained from the different culture media, as evaluated via Tukey’s HSD test (*p* < 0.05) applied after the one-way ANOVA.

**Figure 2:**
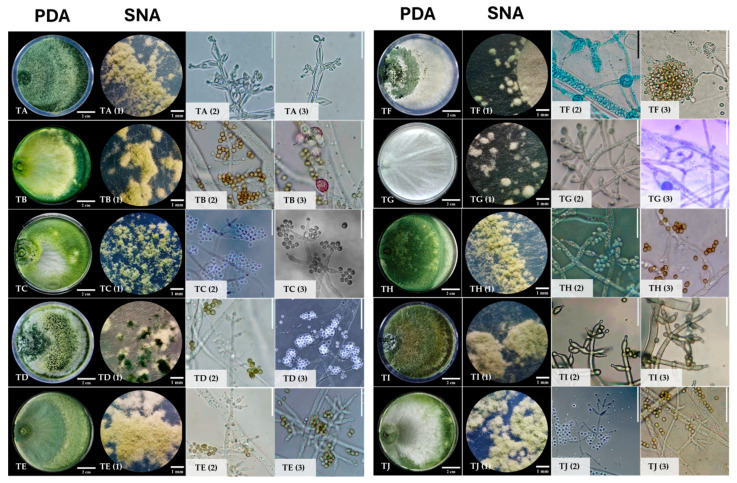
Morphological features of *Trichoderma* TAS. (TA-TJ) Colony morphology grown on PDA media after 7 days of incubation at 27 °C. TA-TJ (1) Pustules developed on SNA media, visualized under a stereomicroscope. TA-TJ (2–3) Microscopic structures (spores, hyphae, conidiophores, chlamydospores, and phialides). Microscopic structures were observed at 100× magnification. Scale bar = 20 µm.

The present study focused on the taxonomic identification of ten root-derived isolates pertaining to the genus *Trichoderma* (TA to TJ). Colony morphology was assessed on three different media: PDA, CMD, and SNA. Most of the isolates exhibited concentric ring formation, sporulation in pustules and tufts, and the presence of diffuse mycelium on the CMD and SNA. In contrast, the PDA supported more robust colonies with abundant aerial mycelium and more profuse sporulation (Figure 1A and Supplementary Table S1). The colony radius was measured 72 h post-inoculation (hpi) (Figure 1B). The comparative analysis conducted revealed that medium type significantly influences colony radial growth. The colonies grown on PDA exhibited the largest average radius, followed by those grown on CMD and SNA. In the PDA colonies, most strains reached a colony radius of approximately 75 mm, except TA, TF, TH, and TI. At the same time, all isolates fully covered the plate by 96 hpi, demonstrating that PDA promotes both mycelial development and sporulation. While CMD supported moderate growth (~65 mm) in most strains, TF was significantly affected. In contrast, SNA was the most growth-restrictive medium, with a colony radius of around 60 mm found for nearly all isolates. The physical appearance and color of the colony, as well as the aggregation of spores in pustules, were observed on both the PDA and SNA media after the seventh day of the *Trichoderma* culture grown at 27 °C (Figure 2). The strains were found to present colors ranging from green to dark green and greenish to pale yellow, as well as a range of white colors. The color of the conidia changed from white to yellowish during the first three days of incubation. The microscopic characteristics observed included structures typical of the genus *Trichoderma*, such as conidiophores bearing phialides and clusters of conidia, while strains TB, TF, and TG also produced chlamydospores (Figure 2). The conidia were globose to subglobose, measuring 1.31–1.36 μm on average. The length of the phialide was 10.48–11.6 μm. All measurements of these structures are summarized in Supplementary Table S2, which compiles the morphological and microscopic characteristics corresponding to the present study.

### 3.2. Molecular Identification and Phylogenetic Analysis

**Figure 3:**
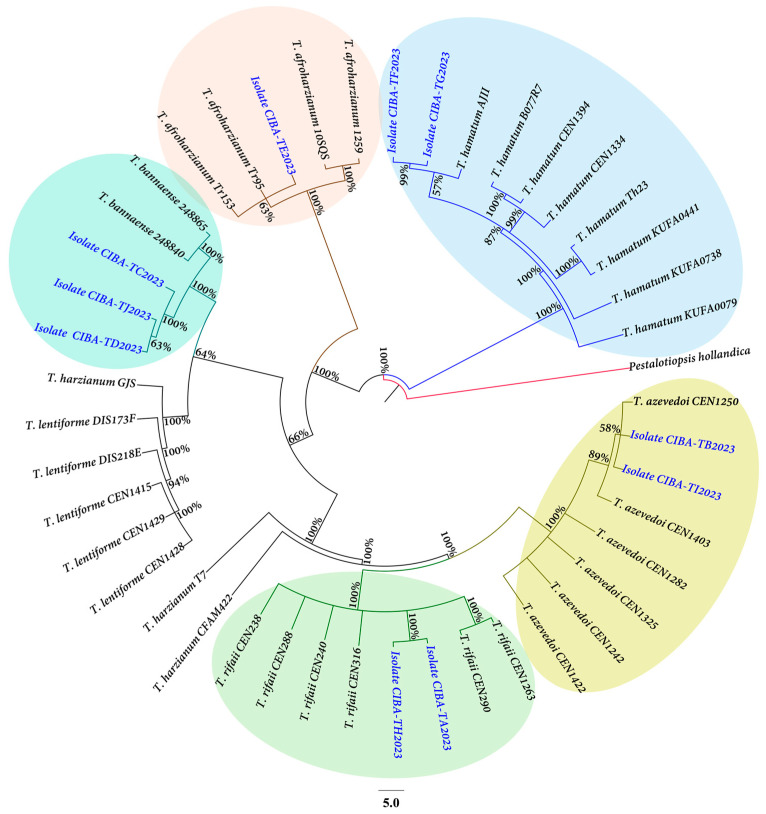
Bayesian phylogenetic tree based on concatenated sequences (ITS, TEF1-α, and RPB2) and showing the relationships between the isolates obtained by the present study and species of the genus *Trichoderma*. *Pestalotiopsis hollandica* was used as the outgroup, with the GenBank accession numbers MH855436.1 (ITS), MH554936.1 (RPB2), and KM199481.1 (TEF1-α). The percentages at the nodes represent posterior probabilities. Different colors represent the clades.

Preliminary sequence similarity searches conducted on the ITS regions confirmed that all isolates belonged to the genus *Trichoderma*, with similarity percentages exceeding 90% in each case. Based on this identification, a phylogenetic analysis was conducted using a concatenated multilocus dataset (comprising ITS, TEF1-α, and RPB2), which revealed the presence of five distinct species within the genus. In the resulting phylogenetic tree (Figure 3), isolates TH and TA clustered closely with *T. rifaii*, while TC, TD, and TJ clustered with *T. bannaense*-related taxa. Isolate TE showed affinity with *T. afroharzianum*, TF and TG grouped with *T. hamatum*, and, finally, TB and TI clustered with *T. azevedoi*. The alignment comprised 45 taxa, including the outgroup.

### 3.3. In Vitro Screening for Plant Growth Promotion Activities

The results obtained for the different PGP traits evaluated demonstrated significant differences between the species and isolates (*p* < 0.05) (Table 2). The highest phosphate solubilization (IPS) was presented by *T. harzianum* (TH3) (55.33 µg/mL), followed by *T. rifaii* (TH) and *Trichoderma* sp. (TJ). At the same time, the lowest values corresponded to *T. atroviride* (Twt) and *T. hamatum* (TF). Regarding the OAPI, *T. azevedoi* (TI) presented the highest value (2.98), while *T. harzianum* (TH3) showed the lowest (0.66). The SPI showed clear differences among the isolates, with *Trichoderma* sp. (TJ) reaching the highest value, followed by *T. hamatum* (TF), while *T. asperellum* (TASP) showed the lowest (1.47). In terms of IAA production, the highest levels were observed in *T. hamatum* (TG) and *T. atroviride* (TWT) (66.45 and 61.21 µg/mL, respectively), followed by *T. hamatum* (TF) and *T. azevedoi* (TI), while the lowest values corresponded to *Trichoderma* sp. (TD and TC, respectively). Cultures grown without L-tryptophan supplementation produced negligible levels of IAA and were therefore excluded from the main table for clarity. These data are provided in Supplementary Table S3.

**Table 2:** PGP activities of *Trichoderma* spp.

Species/Isolated	IPS (µg/mL)	OAPI	SPI	IAA (µg/mL)
*T. rifaii* (TA)	32.00 ± 3.2 cd	2.55 ± 0.11 ab	1.88 ± 0.11 bc	10.59 ± 1.17 fg
*T. azevedoi* (TB)	20.97 ± 0.65 fg	1.94 ± 0.22 bcde	2.20 ± 0.26 abc	19.54 ± 3.77 ef
*Trichoderma* sp. (TC)	31.04 ± 3.42 cd	1.53 ± 0.12 cdef	1.77 ± 0.27 bc	3.49 ± 1.39 g
*Trichoderma* sp. (TD)	33.51 ± 0.45 c	2.16 ± 0.26 bcd	2.45 ± 0.38 abc	2.87 ± 1.87 g
*T. afroharzianum* (TE)	23.93 ± 1.62 ef	1.97 ± 0.25 bcde	1.86 ± 0.05 bc	10.13 ± 0.71 fg
*T. hamatum* (TF)	18.71 ± 0.94 g	1.55 ± 0.06 cdef	2.91 ± 0.08 ab	49.32 ± 0.93 b
*T. hamatum* (TG)	20.31 ± 0.50 fg	2.36 ± 0.40 abc	2.58 ± 0.30 abc	**66.45 ± 7.71 a**
*T. rifaii* (TH)	47.62 ± 0.87 b	1.44 ± 0.47 defg	2.25 ± 0.23 abc	10.13 ± 0.53 fg
*T. azevedoi* (TI)	27.62 ± 0.72 de	**2.98 ± 0.06 a**	2.65 ± 0.70 abc	43.46 ± 3.35 bc
*Trichoderma* sp. (TJ)	45.87 ± 0.98 b	1.92 ± 0.38 bcde	**3.31 ± 0.51 a**	13.83 ± 3.08 f
*T. harzianum* (TH3)	**55.33 ± 1.11 a**	0.66 ± 0.17 g	2.15 ± 0.60 abc	31.27 ± 0.46 d
*T. koningiopsis* (TK11)	30.71 ± 0.77 cd	1.30 ± 0.13 efg	1.90 ± 0.08 bc	34.51 ± 4.24 cd
*T. atroviride* (TWT)	16.34 ± 1.68 g	1.10 ± 0.17 fg	2.63 ± 0.53 abc	**61.21 ± 4.26 a**
*T. asperellum* (TASP)	19.63 ± 1.75 fg	1.97 ± 0.21 bcde	1.47 ± 0.10 c	26.02 ± 6.59 de

Data are presented as mean value ± standard deviation of three replicates. IPS (inorganic phosphate solubilization), OAPI (organic acid production index), SPI (siderophore production index), and IAA (indole-3-acetic acid production). Values with different letters indicate statistically significant differences among treatments as determined by Duncan’s test (*p* < 0.05) applied after one-way ANOVA. Letters in bold indicate the highest value for each variable.

### 3.4. Impact of Trichoderma *spp.* on Maize Seedling Growth

**Figure 4:**
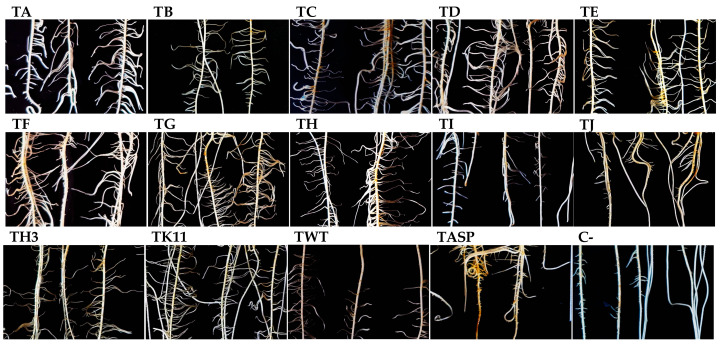
Effect of seed growth promotion conducted with *Trichoderma* spp. on the root architecture of seven-day-old maize seedlings. In the upper left corner of each panel, the treatments are abbreviated as follows: *Trichoderma* TAS (TA–TJ); *Trichoderma* non-TAS (TH3–TASP); and control without fungus (C-).

The early development of maize seedlings showed a general trend toward improved root growth (Table 3). First, all evaluated strains, except for TJ and TWT, promoted a significant increase in root length compared to control plants. The greatest root lengths (≥12 cm) were recorded in seedlings inoculated with TH3, TD, TA, TH, and TG; however, these values did not differ statistically from those obtained with the other strains. The highest value in root dry biomass was observed in seedlings treated with TF. Nevertheless, this value did not differ significantly from most treatments within the TAS group, except for TA, the non-TAS strains (TH3, TK11), and the control, which exhibited lower values. Finally, the highest number of lateral roots (Figure 4) was recorded in TG, TF, and TH belonging to TAS (Table 3). The remaining treatments did not show significant differences compared to the control (7.2 ± 2.6 roots per cm).

Regarding shoot length, the highest values were recorded in treatments TF and TH3, although these differences were not statistically significant compared to most of the evaluated treatments (TA, TB, TD, TE, TG, TH, TI, TK11, or the control). For shoot dry weight, no significant differences were detected among most treatments. The highest shoot dry weight value was obtained in plants treated with TI and was only significantly reduced in plants treated with TK11.

*Trichoderma hamatum* (TF) stands out as a PGPF by displaying the highest values in shoot length and root dry weight. *T. hamatum* (TG) also stands out by showing the highest values in root length and number of secondary roots, along with high values in root dry weight. These changes in root architecture correlate with the fact that this strain exhibits the highest level of IAA production (Table 2). *T. azevedoi* (TI) results on shoot biomass correlate with the highest values on OAPI (Table 2).

### 3.5. In Vitro Screening of Antagonistic Activity of Trichoderma *spp.*

#### 3.5.1. Antagonism of *Trichoderma* spp. Against Phytopathogens in Dual Culture

**Figure 5:**
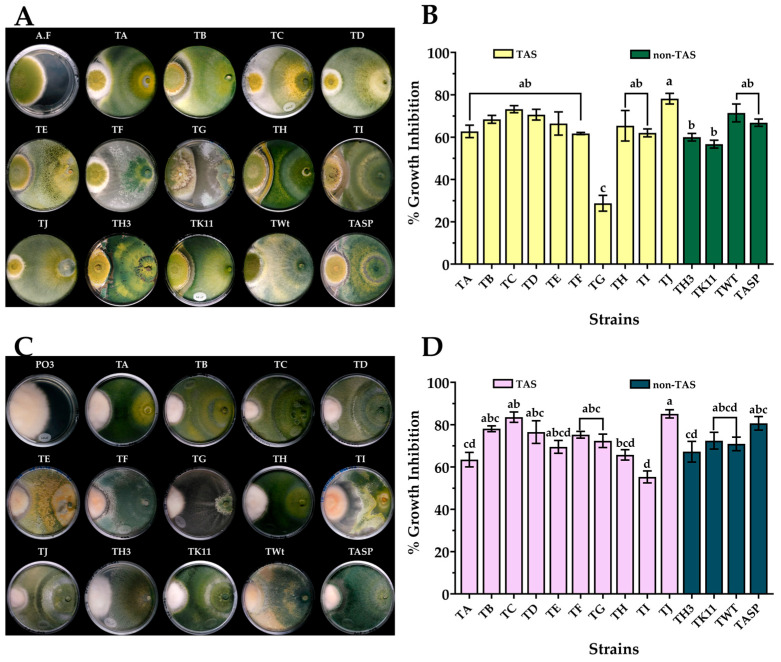
Antagonistic activity of *Trichoderma* spp. against *A. flavus* and *F. verticillioides*. (**A**,**C**) Dual confrontation assays on PDA after seven days of incubation at 25 °C. *Trichoderma* spp. colonies are positioned on the right side of each Petri dish, while *A. flavus* and *F. verticillioides* are found on the left. (**B**,**D**) Phytopathogen inhibition percentage index (%). Treatments are categorized as TAS or non-TAS isolates. Error bars represent standard deviation (SD), and different letters indicate statistically significant differences among treatments according to Tukey’s test (*p* < 0.05) applied after a one-way ANOVA.

All fourteen *Trichoderma* spp. isolates reduced pathogen mycelial growth, with inhibition percentages ranging from 60% to 80% (Figure 5). The highest inhibition of *A. flavus* (78.1%) was observed for isolate TJ, which was statistically similar to most TAS isolates except for TG and the non-TAS isolates TH3 and TK11 (Figure 5A,B). A similar trend was found for *F. verticillioides*: TJ showed the highest inhibition value (85.1%), comparable to most isolates except for TA, TH, TI, and TH3, which exhibited inhibition levels below 60% (Figure 5C,D). TJ showed in dual-culture assays the best growth inhibition response against both fungal phytopathogens. 

Two types of antagonism were observed to be exerted on *A. flavus*, with the first being contact inhibition. This first type corresponded to the majority of the treatments applied, which limited the growth of the phytopathogen. Some of these strains (TA, TC, TD, TF, TJ, and TASP) also impeded maturation and sporulation, resulting in yellow colonies with whitish borders and growth reduction compared to the control (Figure 5A). Remote inhibition, the second type of antagonism, was observed in the remaining treatments (TB, TE, TH, TI, and TK11).

For *F. verticillioides*, contact inhibition was observed in all treatments except TI. This strain, together with TA, TG, and TH, exhibited remote inhibition. However, *F. verticillioides* also appeared to affect the growth of these strains, as their colonies were smaller than those of the control. Interestingly, the antagonistic activity of some of the strains, such as TI, TE, TF, and TwT, led to the orange pigmentation observed in the phytopathogenic colony, in contrast to the other treatments (Figure 5C).

#### 3.5.2. Inhibition of Fungal Phytopathogens by VOCs and Antibiosis Produced by *Trichoderma* spp.

**Figure 6:**
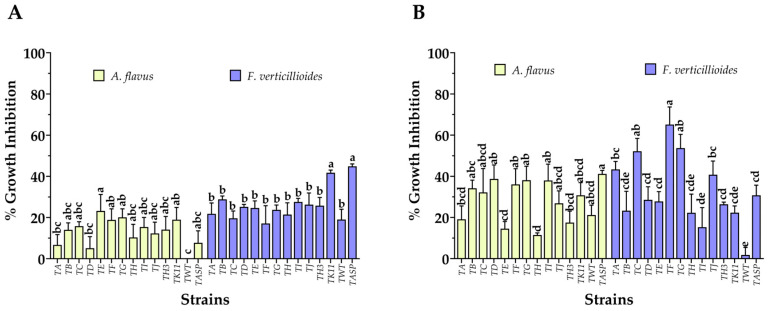
Growth inhibition of the two phytopathogens by volatile (VOC) and non-volatile (antibiosis) compounds produced by *Trichoderma* spp. (**A**) Percentage of growth inhibition mediated by *Trichoderma* VOCs. (**B**) Percentage of growth inhibition mediated by *Trichoderma* antibiosis in dual-confrontation assays. Values represent the mean ± standard deviation (SD) (*n* = 3). Both assays were performed on PDA medium for 72 h at 27 °C. Different letters indicate significant differences among treatments according to Duncan’s test (*p* < 0.05) following a one-way ANOVA.

During the VOC assay (Figure 6A), strain TE showed the highest inhibition of *A. flavus*, although it did not differ significantly from most strains, except for TA, TD, and TWT. In contrast, non-TAS TK11 and TASP exhibited significantly greater inhibition of *F. verticillioides* growth compared with the rest of the strains. Regarding the antibiosis assay (Figure 6B), strain TASP showed the highest inhibition of *A. flavus*, although it did not differ significantly from most strains, except for TA, TE, TH, and TH3. On the other hand, *F. verticillioides* was most strongly inhibited by strains TF, TG, and TC, with no significant differences among them; the remaining strains showed inhibition values below 40% through antibiosis.

**Figure 7:**
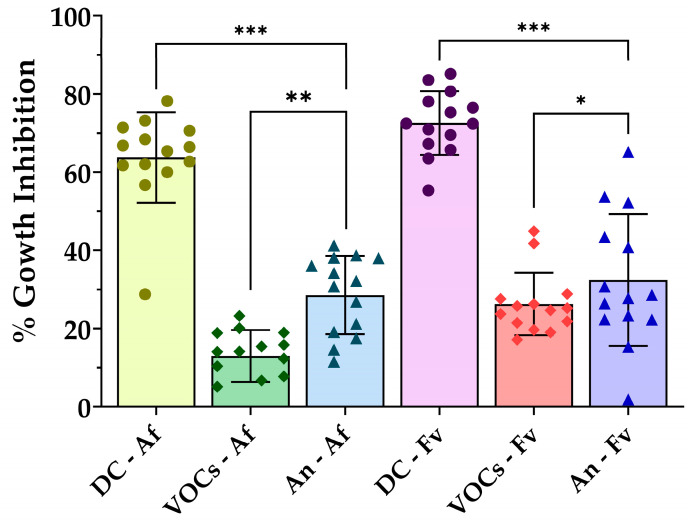
Percentage of growth inhibition of *A. flavus* and *F. verticillioides* under three treatment conditions: dual-culture (DC), volatile organic compound (VOC), and antibiosis (An). Values represent means ± standard deviation. Significant differences among treatments were determined by means of a one-way ANOVA followed by Tukey’s HSD test. Significance levels indicated as *p* < 0.05 (*), *p* < 0.01 (**), and *p* < 0.001 (***).

The global antagonism analysis (Figure 7) showed that the dual-culture (DC) assay exhibited the highest levels of growth inhibition against both phytopathogens, particularly against *F. verticillioides*. Inhibition rates differed significantly among the evaluated mechanisms (ANOVA followed by Tukey’s HSD test, *p* ≤ 0.05). The DC assay reached mean inhibition values of approximately 65–70% for *A. flavus* and 80% for *F. verticillioides*, whereas antibiosis showed intermediate inhibition levels (~35–45%), and VOC-mediated effects resulted in the lowest inhibition (~20–25%). Although both VOC and antibiosis assays exhibited significantly lower inhibition than the DC assay, measurable antagonistic effects were still observed. Overall, a differential response of the phytopathogens to the evaluated functional activities was evident, with *F. verticillioides* consistently showing greater susceptibility to *Trichoderma* spp. than *A. flavus*.

### 3.6. Partial Least Squares Discriminant Analysis

**Figure 8:**
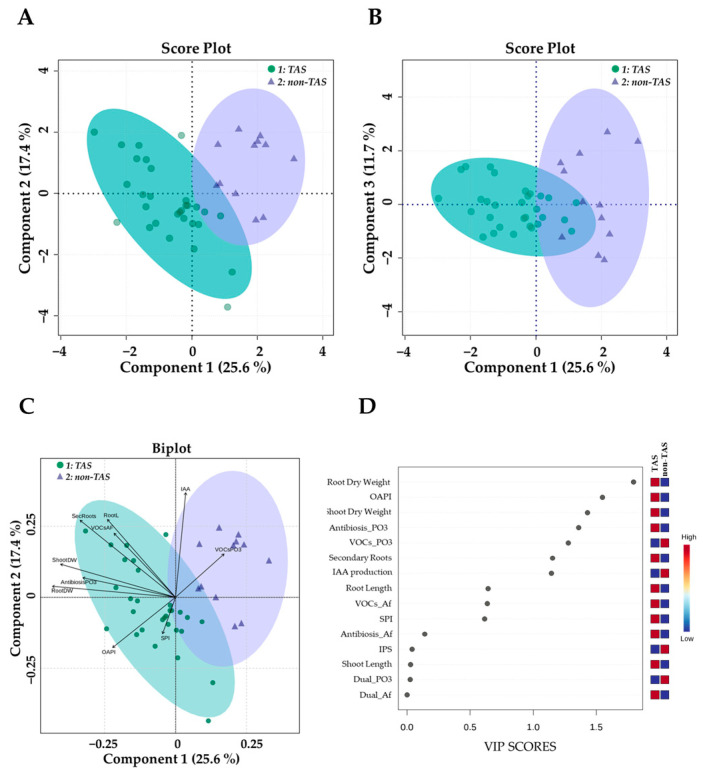
PLS-DA of *Trichoderma* TAS and non-TAS. (**A**,**B**) Score plots showing group separation across the main components. (**C**) Biplot illustrating variable loadings in relation to strain distribution. (**D**) VIP scores identifying the traits contributing most to class discrimination.

A partial least squares discriminant analysis (PLS-DA) was performed to investigate multivariate patterns distinguishing teosinte-associated strains (TAS) from non-teosinte-associated strains (non-TAS) and identify the variables contributing most strongly to this separation (Figure 8). The model explained 68.8% of the total variance across five components, with the first three components accounting for 54.7% of the variation. The model showed satisfactory explanatory capacity (R^2^ = 0.73) and moderate predictive ability (Q^2^ = 0.55). To evaluate the model’s robust predictive power and rule out overfitting, a permutation test (*n* = 100) was performed, confirming that the predictive reliability of the model was highly significant (*p* < 0.01) (Supplementary Figure S1). This statistical validation demonstrates that the observed discrimination is driven by genuine, structured biological variation rather than random separation. Score plots based on Component 1 vs. Component 2 (Figure 8A) revealed a statistically validated separation between TAS (green circles) and non-TAS (purple triangles), with minimal overlap between confidence ellipses. A comparable clustering pattern was observed in the Component 1 vs. Component 3 score plot (Figure 8B), although with slightly increased overlap, suggesting that the primary discrimination is largely driven by the first component. These results demonstrate a consistent grouping of isolates according to their origin and functional traits. The corresponding biplot (Figure 8C) provided insight into the variables driving this separation. Traits associated with plant growth promotion, such as root and shoot dry biomass, lateral root number, and indole-3-acetic acid (IAA) production, were oriented toward the TAS cluster, indicating a strong contribution to this group. In contrast, variables related to antagonistic activity, including antibiosis and VOC-mediated inhibition (particularly against *Fusarium verticillioides* PO3), were more strongly associated with the non-TAS isolates. This distribution highlights a functional differentiation between groups, where TAS isolates are primarily linked to plant growth promotion, while certain non-TAS isolates show enhanced biocontrol-related traits. The variable importance in projection (VIP) analysis (Figure 8D) further supported these patterns, identifying root dry biomass, organic acid production index (OAPI), shoot dry biomass, antibiosis, and VOC-mediated inhibition against *F. verticillioides* PO3 as the most influential variables (VIP > 1). Additional important contributors included lateral root number, IAA production, and root length. These results indicate that both growth-related and antagonistic traits play a key role in structuring the multivariate differentiation among the isolates.

**Figure 9:**
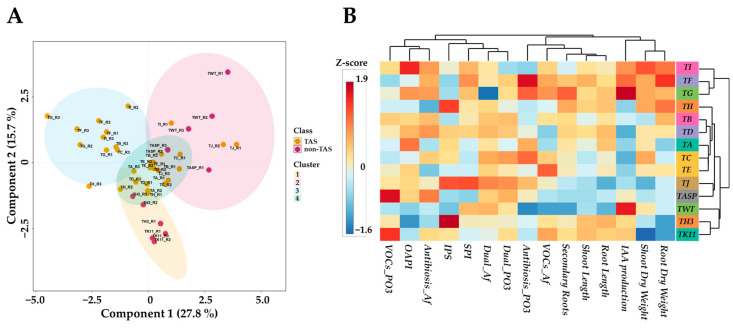
The multivariate functional performance of the *Trichoderma* sp. (**A**) PCA–k-means clustering and hierarchical heatmap of Z-score-standardized functional traits across *Trichoderma* strains. The letter R followed by a numeral refers to the biological replicate per isolate (three replicates per strain). (**B**) Heatmap illustrating strain-level patterns in plant growth promotion and biocontrol variables.

Figure 9 provides an integrated view of the multivariate functional performance of the *Trichoderma* sp. isolates by combining PCA–k-means clustering and a heatmap with a hierarchical dendrogram, which together allow the visualization of similarity patterns and the formation of functional clusters. The PCA-k-means analysis (Figure 9A) summarizes the multivariate structure of the isolate dataset, showing that the first two principal components explain 43.5% of the total variation and clearly separate the strains into four functional classes. Group 1 (mainly TH3 and TK11) and Group 2 (mainly TWT, TASP, and TJ) are composed mostly of non-TAS isolates, whereas Groups 3 (TG, TF, and TI) and 4 (the remaining isolates) correspond to TAS isolates. Groups 3, 1, and 2 show greater functional differentiation according to their dispersion in the PCA space, while Group 4 is in the central region, reflecting a moderate and similar functional performance among its members. The heatmap (Figure 9B) corroborates the functional structure revealed by the PCA-k-means analysis, showing well-defined gradients in the standardized values of each trait. The isolates in Group 3 (TG, TF, and TI) exhibit the highest Z-scores in parameters associated with plant growth promotion, including shoot and root dry weight, IAA production, and root traits, whereas within Group 2, strain TJ stands out for displaying the highest values in antagonistic variables, particularly antibiosis and dual confrontation against both phytopathogens. Consistent with their intermediate position in the PCA, TK11, TH3, and TASP show a mixed pattern, with moderate PGP values and strong VOC-mediated inhibition. In contrast, isolates in Group 4 maintain balanced profiles without marked extremes, which aligns with their central location in the PCA space. Overall, the heatmap reinforces the functional differentiation among the groups identified in the PCA, validating the promoter, antagonist, and intermediate tendencies observed at the individual level.

## 4. Discussion

### 4.1. Isolation and Identification

Various studies have demonstrated that the teosinte rhizosphere harbors a diverse microbial community that performs key ecological functions, contributing to plant resilience and agricultural sustainability [4,5,6]. The study of beneficial microorganisms in wild species such as *Zea mays* spp. *mexicana* provides a pathway to rediscovering functional relationships that may have been attenuated during maize domestication [1,7]. Research on the teosinte rhizosphere has reported the presence of bacterial genera, such as *Pseudomonas*, *Bacillus*, and *Enterobacter*, that can solubilize nutrients and produce siderophores, phytohormones, and antimicrobial compounds. These microorganisms also induce systemic plant resistance [1,3,7].

Furthermore, studies have identified bacterial endophytes, such as *Rhizobium* and *Stenotrophomonas*, that can establish non-nodulating symbioses with wild grasses and adapt to adverse conditions [61]. These findings suggest that teosinte conserves an ancestral microbiome with a high level of biotechnological potential, which is useful for developing bioinoculants adapted to local agroecological systems [1,2]. In this context, the search for *Trichoderma* TAS from the rhizosphere is strategic, given that this ancestral niche may host unique functional fungal strains adapted to specific conditions with great potential as resilient bioinoculants [15,62].

The present study identified most isolates at the species level using an integrative approach combining morphological characteristics and multilocus sequence data. However, three isolates (TC, TD, and TJ) were assigned to *Trichoderma* sp. aff. *bannaense*, indicating a close but unresolved phylogenetic relationship. This conservative designation is consistent with current taxonomic practice when multilocus support is incomplete [31]. The limited resolution is likely associated with the reduced discriminatory power of ITS and TEF1α within closely related *Trichoderma* clades, together with the absence of RPB2 sequences and the limited availability of reference data for *T. bannaense* in public databases [27,31]. Further studies incorporating additional loci and expanded reference datasets, combined with complementary morphological characterization, will be necessary to clarify the taxonomic status of strain TJ and determine whether it represents intraspecific variation or a potentially uncharacterized taxon.

### 4.2. PGPF Activity

Once the *Trichoderma* TAS were identified, various PGPF traits were compared with those of previously characterized reference *Trichoderma* non-TAS (*T. harzianum*, *T. atroviride*, *T. asperellum*, and *T. koningiopsis*), given their efficacy as biocontrol agents and plant growth promoters. This comparison enabled both the establishment of reference parameters and the identification of the possible adaptive advantages of the local isolates.

*Trichoderma hamatum* (TF) stands out as a PGPF by displaying the highest values in shoot length and root dry weight. *T. hamatum* (TG) also stands out by showing the highest values in root length and number of secondary roots, along with high values in root dry weight. These changes in root architecture correlate with the fact that this strain exhibits the highest level of IAA production (Table 2). TG also shows high production of organic acids. The *T. azevedoi* (TI) results on shoot biomass correlate with the highest values on OAPI, IAA, and siderophores (Table 2). *Trichoderma* solubilizes phosphate through the production of organic acids [63], which was confirmed in this study. However, differences between solubilization values obtained under submerged fermentation and the OAPI values indicate that acidification alone does not fully explain the variability observed. This suggests the involvement of additional factors, such as the secretion of phosphatases and phytases [64,65,66]. Additionally, the PGPF activity associated with siderophores can be explained by their chelating function; they have a high affinity for iron, which is indispensable for photosynthesis, cellular respiration, and the functioning of various redox enzymes in plants [67,68,69,70]. This capacity not only enhances plant nutrition but also strengthens microbial competition in the rhizosphere by reducing iron availability for phytopathogens [71,72,73]. There are reports of siderophore production in *T. hamatum* [73,74,75], *T. asperellum* [71,76], *T. atroviride* [15], and *T. virens* [77], all of which have been associated with biocontrol and plant growth promotion. Nevertheless, our results from the biochemical assays and PGPF activity in maize seedlings indicate that lesser-studied species may exhibit equal or even greater potential under specific experimental conditions. Regarding the PGPF activity associated with IAA production, this can be explained by the regulatory role that this metabolite plays in essential functions related to morphogenesis and cell division [78]. It is the principal auxin responsible for modulating root system architecture and is associated with plant growth, lateral root formation, and tissue differentiation processes [78,79]. Some pathogens have been reported to produce it as a virulence mechanism since alterations in hormonal signaling favor colonization and symptom development [80]. However, IAA synthesis by *Trichoderma* has been associated with positive effects on root architecture and plant growth promotion [81,82]. The IAA levels obtained (2.87–66.45 µg/mL) fall within the average range reported for other species (*T. harzianum*, *T. asperellum*, *T. viride*, among others), although they remain below the maximum values (90–118 µg/mL) reported in the literature [83,84].

The remaining strains showed limited or no PGPF activity in the evaluated parameters. Their performance in the biochemical assays was also moderate or low, which could account for their reduced effectiveness as plant growth promoters in seedlings. These results are consistent with the literature reporting the ability of *T. hamatum* (TF and TG in this study) to stimulate shoot biomass and enhance plant vigor [10,19,78,85,86]. The stimulation of root growth and vigor by these strains is particularly relevant, as longer, more robust roots and greater root density facilitate soil exploration and nutrient uptake, in addition to increasing tolerance to adverse biotic and abiotic stress conditions [78,79,81]. Beyond classical mechanisms such as phytohormone production, nutrient solubilization, and antibiosis, it has been reported that *Trichoderma* spp. can actively modulate plant immune responses to facilitate colonization. This involves molecular strategies such as the secretion of effectors, interference with host signaling pathways, and the production of regulatory molecules, enabling the establishment of beneficial plant–fungus interactions [87]. Therefore, further research under plant-associated conditions will be necessary to determine whether the observed traits are effectively expressed during interaction with maize plants.

### 4.3. BCA Activity

The main mechanisms that confer *Trichoderma* its biocontrol capacity are mycoparasitism, antibiosis, and competition for space and nutrients, processes regulated by its versatile metabolism, production of lytic enzymes, synthesis of secondary metabolites, and resistance to microbial inhibitors [10,88]. These mechanisms provide *Trichoderma* with a marked competitive advantage over phytopathogenic fungi during rhizosphere colonization [89]. The dual culture assay is an efficient technique widely used for the in vitro selection of effective *Trichoderma* strains for the biocontrol of various phytopathogenic fungi [90,91,92,93]. According to the multivariate (PLSDA) analysis and hierarchical clustering of the *Trichoderma* isolates, the strain *Trichoderma* sp. (TJ) is considered the most effective antagonist, as it exhibited strong inhibitory activity in dual-culture assays against both phytopathogens, in addition to showing high antibiosis values against *F. verticillioides* and the best performance in siderophore production. This may be related to the fact that siderophores are used by fungi during nutrient competition mainly for iron, and TJ may mediate its antagonistic activity through this mechanism as well as through direct interaction with the phytopathogens [71,72,73]. Other strains that also stand out for their antagonistic activity are the *T. hamatum* isolates (TG and TF), which displayed the highest antibiosis values against *F. verticillioides* and moderate inhibition through both antibiosis and VOCs against *A. flavus.* This indicates that their antagonistic mechanisms are largely mediated by both diffusible and volatile metabolites, and, specifically in the case of TG, this may be explained by its moderate production of organic acids, which may play a key role in its antagonistic activity by modifying pH and saturating the space with compounds that limit pathogen development. The inhibition observed in *A. flavus* may be explained by conidial immaturity, as *Trichoderma* spp. have been shown to delay conidial maturation in response to antifungal metabolites or nutrient competition [94,95,96]. Likewise, Saldaña–Mendoza et al. [72] demonstrated that nutrient competition is a critical mechanism that delays pathogen maturation. In contrast, pigmentation observed in *F. verticillioides* colonies does not necessarily indicate immaturity as it has also been linked with virulence, secondary metabolite production (including phytotoxic pigments), biotic stress, or degraded metabolites [97,98]. In our assays, this phenotypic observation may reflect biotic stress during interaction with *Trichoderma.* However, further molecular and chemical analyses are necessary to establish clear functional associations. Other strains with notable but more specific results include *T. afroharzianum* (TE), which showed the highest VOC-mediated inhibition of *A. flavus*, and *T. asperellum* (TASP) and *T. koningiopsis* (TK11), which exhibited the highest VOC-mediated inhibition of FPO3. The remaining strains exhibited moderate to low antagonistic activity across the different assays. Voloshchuk et al. [94] reported that *Trichoderma* spp. achieved inhibition percentages above 60% against *A. flavus* and *A. parasiticus*, mainly through antifungal metabolites. In contrast, Jambhulkar et al. [98,99] reported inhibition levels of 81–87% for *F. verticillioides*, indicating greater sensitivity of this species compared to *A. flavus*, which aligns with the values obtained in the present study. Previous studies have consistently shown that VOCs produced by *Trichoderma* can inhibit several important phytopathogens, with reported effects ranging from ~24% to ~56% in *Fusarium* spp. and up to ~50% in *Aspergillus* spp. [94,96,99,100].

Together, supervised (PLS-DA) and unsupervised (PCA–k-means) multivariate approaches (Figure 8 and Figure 9) provided complementary perspectives on the functional performance of the *Trichoderma* isolates, revealing a clear differentiation between TAS strains and non-TAS controls. Importantly, this separation reflects not only statistical clustering but also functional divergence, with TAS isolates generally displaying more consistent and multifunctional profiles, particularly in traits associated with plant growth promotion, compared to the more variable or specialized responses observed in non-TAS strains. Within the TAS group, TG, TF, and TI emerged as the most consistent candidates for root phytostimulation, while TJ exhibited strong antagonistic properties, particularly through dual-culture inhibition of both phytopathogens. Other isolates showed intermediate performance, reflecting the intrinsic diversity of the teosinte rhizosphere microbiome. Collectively, these results reinforce the role of the teosinte (Shushu-like) rhizosphere as a unique ecological reservoir of *Trichoderma* diversity with enhanced biocontrol and plant growth-promoting capacities [4,5,6]. This distinctive functional profile may be explained by the ancestral ecological context of teosinte, where reduced anthropogenic pressure and long-term plant–microbe coevolution have favored the selection of resilient and multifunctional microorganisms [1,2,6]. Such environments promote the maintenance of complex microbial communities capable of simultaneously supporting plant growth and suppressing pathogens, thereby conferring adaptive advantages to TAS strains over non-native isolates. Importantly, previous studies have demonstrated that beneficial microorganisms derived from ancestral habitats can be successfully transferred to modern maize systems, both in bacterial and fungal contexts [7,101], supporting the feasibility of applying TAS isolates in contemporary agroecosystems. Expanding research in this direction will help to clarify the extent to which teosinte-derived *Trichoderma* can serve as multifunctional bioinoculants, effectively bridging ecological heritage with modern crop improvement strategies.

## 5. Conclusions

Five different *Trichoderma* species—*T. rifaii*, *T. azevedoi*, *T. afroharzianum*, *T. hamatum*, and *Trichoderma* sp. (aff. *bannaense*)—were found to be associated with the teosinte rhizosphere. The TAS *Trichoderma* strains of interest exhibited superior performance across most traits evaluated, particularly those associated with antagonistic activity and root development stimulation. Specifically, *Trichoderma* sp. (aff. *bannaense*) (TJ) and *T. hamatum* (TF, TG) demonstrated the highest functional potential as biocontrol and biostimulant agents. Therefore, these strains are promising candidates for the development of targeted bioinoculants to enhance maize health and productivity under locally adapted agricultural conditions.

While the present study provides valuable insights into the functional potential of *Trichoderma* strains associated with the teosinte rhizosphere, several limitations should be acknowledged. The experimental approach was primarily based on in vitro assays and early-stage seedling evaluations, which, although effective for initial screening, do not fully represent the complexity of field conditions, where interactions with indigenous microbiota and environmental variability may influence strain performance. Additionally, the ability of teosinte-associated strains to establish and persist in the rhizosphere of modern maize hybrids remains to be demonstrated, as potential host-specific adaptations may affect their effectiveness across different cropping systems. Furthermore, the lack of detailed soil physicochemical characterization at the sampling sites, including parameters such as pH, organic matter content, and soil texture, limits a more comprehensive ecological interpretation of the isolates. Future research should address these aspects through greenhouse and field-based evaluations, as well as through integrated soil analyses, to validate the consistency, persistence, and functional applicability of the most promising *Trichoderma* strains in agricultural systems.

## Figures and Tables

**Table 1:** Identification of *Trichoderma* species and NCBI GenBank accession numbers for ITS, tef*1-α*, and *rpb2* sequences.

Strain Code	Identification	GeneBank Number		
		ITS	*tef1-α*	*rpb2*
CIBATA2023	*T. rifaii*	PX682131	PX943418	PX974252
CIBATB2023	*T. azevedoi*	PX682132	PX943419	PX974253
CIBATC2023	*Trichoderma* sp. aff. *bannaense*	PX682133	PX943420	PX974254
CIBATD2023	*Trichoderma* sp. aff. *bannaense*	PX682134	PX943421	PX974255
CIBATE2023	*T. afroharzianum*	PX682135	PX943422	PX974256
CIBATF2023	*T. hamatum*	PX682136	PX943423	PX974257
CIBATG2023	*T. hamatum*	PX682137	-*	PX974258
CIBATH2023	*T. rifaii*	PX682138	PX943424	-*
CIBATI2023	*T. azevedoi*	PX682139	PX943425	-*
CIBATJ2023	*Trichoderma* sp. aff. *bannaense*	PX682140	PX943426	-*

Sequences that are unavailable in GenBank are indicated by “-*“.

**Table 3:** Plant growth effects of *Trichoderma* spp. on maize seedling development in paper towel assay.

Species/Isolate	Root Length (cm)	Root Dry Weight (g)	Number of Secondary Roots per cm	Shoot Length (cm)	Shoot Dry Weight (g)
*T. rifaii* (TA)	**12.13 ± 2.7 a**	0.011 ± 0.0013 b	9.6 ± 3.9 bc	9.22 ± 3.0 abc	0.029 ± 0.006 ab
*T. azevedoi* (TB)	11.09 ± 2.3 ab	0.024 ± 0.0021 ab	10.31± 3.3 bc	8.91 ± 2.7 abc	0.027 ± 0.010 ab
*Trichoderma* sp. (TC)	10.43 ± 3.7 ab	0.018 ± 0.0041 ab	9.37 ± 4.8 bc	8.23 ± 2.1 bc	0.028 ± 0.011 ab
*Trichoderma* sp. (TD)	**12.30 ± 1.7 a**	0.020 ± 0.0033 ab	10.41 ± 2.9 bc	10.15 ± 2.8 abc	0.030 ± 0.007 ab
*T. afroharzianum* (TE)	10.68 ± 3.2 ab	0.020 ± 0.0032 ab	9.48 ± 4 bc	8.73 ± 2.7 abc	0.030 ± 0.008 ab
*T. hamatum* (TF)	11.21 ± 3.1 ab	**0.030 ± 0.0032 a**	11.18 ± 3.6 abc	**10.9 ± 3.7 a**	0.034 ± 0.012 ab
*T. hamatum* (TG)	**12.07 ± 2.1 a**	0.023 ± 0.0022 ab	**15.04** ± 4.9 **a**	10.13 ± 2.5 abc	0.034 ± 0.009 ab
*T. rifaii* (TH)	**12.12 ± 2.1 a**	0.025 ± 0.0022 ab	12.14 ± 4.41 ab	10.39 ± 3 abc	0.032 ± 0.009 ab
*T. azevedoi* (TI)	10.77 ± 2.5 ab	0.026 ± 0.0027 ab	9.6 ± 3.23 bc	9.84 ± 3.2 abc	**0.037 ± 0.008 a**
*Trichoderma* sp. (TJ)	7.13 ± 3.8 c	0.017 ± 0.0028 ab	7 ± 2.77 c	7.78 ± 2.2 c	0.025 ± 0.008 ab
*T. harzianum* (TH3)	**12.59 ± 1.3 a**	0.010 ± 0.0011 b	9.78 ± 4.4 bc	**10.9 ± 3.5 a**	0.024 ± 0.007 ab
*T. koningiopsis* (TK11)	11.74 ± 3 ab	0.011 ± 0.0021 b	10.71 ± 3.7 abc	10.8 ± 3.3 ab	0.021 ± 0.005 b
*T. atroviride* (TWT)	7.40 ± 3.2 c	0.016 ± 0.0021 ab	8.22 ± 3.5 c	7.91 ± 1.6 c	0.028 ± 0.003 ab
*T. asperellum* (TASP)	9.34 ± 4.4 bc	0.018 ± 0.0032 ab	7.23 ± 2.63 bc	7.80 ± 2.1 c	0.027 ± 0.007 ab
*Control/CT*	7.68 ± 2 c	0.011 ± 0.0017 b	7.2 ± 2.6 c	9.37 ± 1.9 abc	0.025 ± 0.006 ab

Data are presented as mean value ± standard deviation of the three replicates, and each replicate contained six plants. Values followed by various combinations of letters indicate statistically significant differences among treatments, according to Tukey’s test (*p* < 0.05) applied after a one-way ANOVA. Letters in bold indicate the highest value for each variable.

## Data Availability

The original contributions presented in this study are included in the article; further inquiries can be directed to the corresponding authors.

## Supplementary Materials

The following supporting information can be downloaded at https://www.mdpi.com/article/10.3390/jof12060392/s1, Table S1: Morphological and growth features of *Trichoderma* colonies in PDA, CMD, and SNA culture media; Table S2: Microscopic characterization of *Trichoderma* spp. structures on SNA media; Table S3: Production levels of indole-3-acetic acid (IAA) by *Trichoderma* spp.; Figure S1: PLS-DA of TAS and non-TAS *Trichoderma* strains.
