# Supplementary material for: Trichoderma spp. Associated with Teosinte (Zea mays spp. mexicana) Rhizosphere Exhibit Potential Plant Growth-Promoting and Antagonistic Functional Traits
Source: J Fungi (Basel). 2026 May 29;12(6):392. doi: 10.3390/jof12060392 (PMC13301144; doi:10.3390/jof12060392)
Supplement: Supplementary file 1 [file jof-12-00392-s001.zip › Supplementary Figures.pdf]

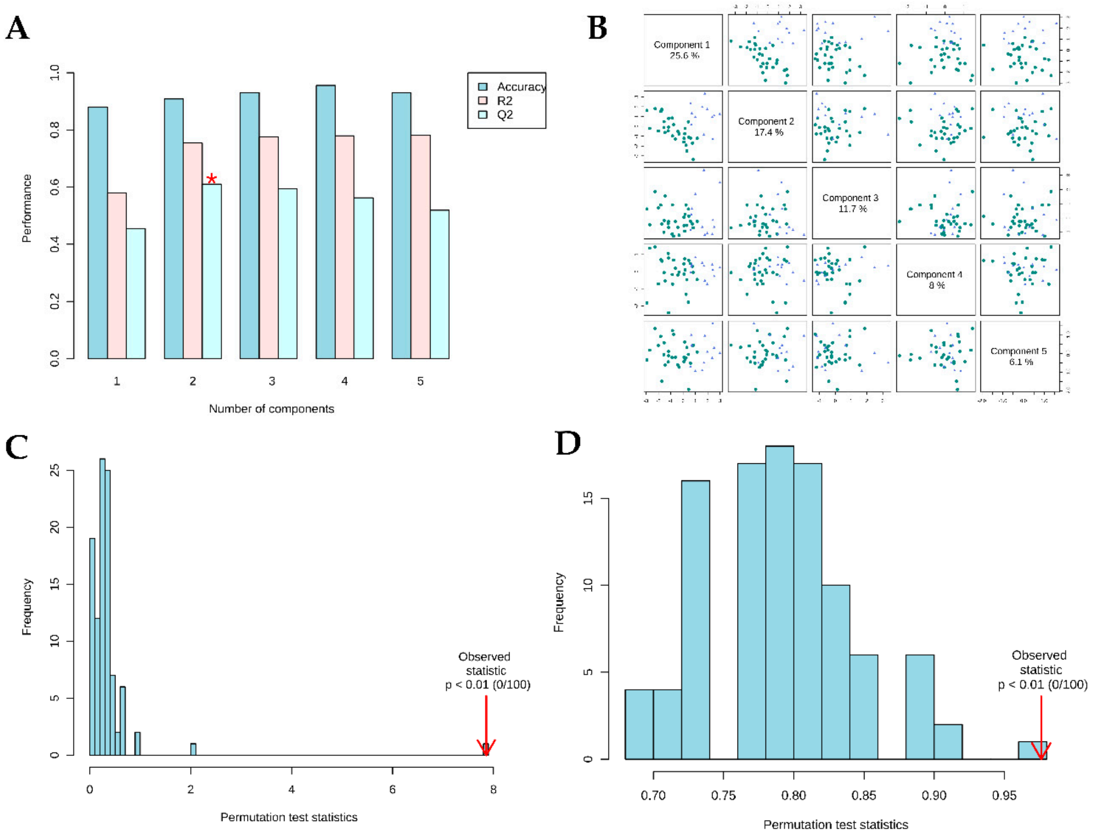

Figure S1. PLS-DA analysis of TAS and non-TAS *Trichoderma* strains
(A) Performance of the PLS-DA model evaluated using Accuracy, R^2, and Q^2 across 1–5 components. The red asterisk indicates the number of components selected as optimal. (B) Pairwise scatter plots showing the distribution of samples for the first five components of the PLS-DA model. The percentages correspond to the variance explained by each component. (C–D) Results of the permutation tests for the PLS-DA model. The histograms display the distribution of permutation test statistics obtained from 100 random permutations of class labels. The red arrows mark the observed statistic for the original model ($p < 0.01$; 0/100).
