# Supplementary material for: Trichoderma spp. Associated with Teosinte (Zea mays spp. mexicana) Rhizosphere Exhibit Potential Plant Growth-Promoting and Antagonistic Functional Traits
Source: J Fungi (Basel). 2026 May 29;12(6):392. doi: 10.3390/jof12060392 (PMC13301144; doi:10.3390/jof12060392)
Supplement: Supplementary file 1 [file jof-12-00392-s001.zip › Supplementary Tables.pdf]

Supplementary Table S1. Morphological and growth features of *Trichoderma* colonies in PDA, CMD, and SNA culture media

Species (Isolate)	Culture media	Texture	Pigments	Pattern of Conidiation
T. rafiai (TA)	PDA	Compact	-	Tufts
	CMD	Granular	-	Diffuse
	SNA	Granular	-	Pustules
T. azevedoi (TB)	PDA	Cottony	+	Tufts
	CMD	Granular	+	Dispersed
	SNA	Granular	-	Dispersed and pustules
Trichoderma sp. (TC)	PDA	Cottony	-	Tufts
	CMD	Granular	-	Dispersed and pustules
	SNA	Granular	-	Granules
Trichoderma sp (TD)	PDA	Cottony	-	Tufts
	CMD	Granular	-	Dispersed and pustules
	SNA	Granular	-	Pustules
T. afroharzianum (TE)	PDA	Compact	-	Tufts
	CMD	Lisa	-	Pustules
	SNA	Granular	-	Granules
T. hamatum (TF)	PDA	Velvet	-	Granules
	CMD	Granular	-	Pustules
	SNA	Granular	-	Pustules
T. hamatum (TG)	PDA	Velvet	-	Granules
	CMD	Granular	-	Pustules
	SNA	Granular	-	Pustules and granules
T. rifaii (TH)	PDA	Compact	-	Tufts
	CMD	Granular	-	Pustules
	SNA	Granular	-	Pustules and granules
T. azevedoi (TI)	PDA	Velvet	+	Tufts
	CMD	Granular	-	Tufts and granules
	SNA	Granular	-	Tufts and granules
Trichoderma sp. (TJ)	PDA	Cottony	+	Tufts
	CMD	Granular	+	Pustules
	SNA	Granular	-	Pustules and granules

Supplementary Table S2. Microscopic characterization of *Trichoderma* spp. structures on SNA media.

Species (Isolate)	Hyphae Width (µm ± SD)	Conidiophore shape and length (µm ± SD)	Chlamydospore type	Conidiophore Branching	Phialide length (µm ± SD)	Conidia Diameter (µm ± SD)
T. rifaii (TA)	2.7 ± 0.8	Straight (20 ± 7.2)	-	Primary and asymmetrical	Ampulliform (6.9 ± 2.3)	Subglobose (2.6 ± 0.2)
T. azevedoi (TB)	4.5 ± 1.7	Curved (15.4 ± 4.2)	Intercalary	Irregular	Ampulliform (7.6 ±1.28)	Globose (2.5 ± 0.21)
Trichoderma sp. (TC)	2.9 ± 0.5	Straight (15.1 ± 4.1)	Terminal	Primary and symmetrical	Ampulliform (6.4 ±1.5)	Globose (2.43 ± 0.23)
Trichoderma sp. (TD)	2.6 ± 0.34	Curved (13.7 ± 3.7)	Intercalary	Primary and symmetrical	Ampulliform (6.5 ±1.2)	Globose (2.3 ± 0.26)
T. afroharzianum (TE)	3.8 ± 0.83	Curved (18.3 ± 3.8)	-	Irregular	Ampulliform (7.3 ± 0.98)	Subglobose (2.4 ± 0.3)
T. hamatum (TF)	3.5 ± 0.82	Straight (17.2 ± 2.5)	Intercalary	Additional and symmetrical	Ampulliform (7.4 ±1.8)	Globose (2.4 ± 0.26)
Trichoderma sp. (TD)	4.3 ± 0.98	Curved (21.6 ± 7.9)	Terminal	Additional and symmetrical	Subulate (12.9 ± 2.5)	Oval (0.9 ± 0.15)
T. rifaii (TH)	4.1 ± 0.61	Curved (25.5 ± 6.5)	-	Irregular	Ampulliform (12.7 ± 2.3)	Subglobose (2.52 ± 0.63)
T. azevedoi (TI)	2.3 ± 0.21	Straight (21.5 ± 4.3)	Terminal	Additional and symmetrical	Ampulliform (8.7 ± 1.7)	Globose (2.3 ± 0.23)
Trichoderma sp. (TJ)	2.3 ± 0.24	Curved (23.5 ± 8.2)	-	Additional and irregular	Ampulliform (8.8 ± 1.2)	Globose (2.5 ± 0.22)

Supplementary Table S3. Production levels of indole-3-acetic acid (IAA) by *Trichoderma* spp.

**Species (Isolate)**	**+IAA (µg/mL)**	**-IAA (µg/mL)**
T. rifaii (TA)	10.59 ± 1.17 fg	ND*
T. azevedoi (TB)	19.54 ± 3.77 ef	1.02 ± 1.1 abc
Trichoderma sp. (TC)	3.49 ± 1.39 g	ND*
Trichoderma sp. (TD)	2.87 ± 1.87 g	1.17 ± 0.46 abc
T. afroharzianum (TE)	10.13 ± 0.71 fg	4.57 ± 0.70 abc
T. hamatum (TF)	49.32 ± 0.93 b	7.34 ± 1.33 a
T. hamatum (TG)	**66.45 ± 7.71 a**	8.12 ± 40 a
T. rifaii (TH)	10.13 ± 0.53 fg	ND*
T. azevedoi (TI)	43.46 ± 3.35 bc	3.79 ± 6.61 abc
Trichoderma sp. (TJ)	13.83 ± 3.08 f	3.02 ± 0.80 abc
T. harzianum (TH3)	31.27 ± 0.46 d	6.26 ± 14.87 ab
T. koningiopsis (TK11)	34.51 ± 4.24 cd	7.19 ± 4.83 a
T. atroviride (TWT)	**61.21 ± 4.26 a**	ND*
T. asperellum (TASP)	26.02 ± 6.59 de	4.26 ± 3.59 abc

*ND: Not detected. +IAA (with L-Trp) and –IAA (without L-Trp). Values represent mean ± standard deviation of three replicates. Different letters indicate statistically significant differences among treatments according to Duncan's test ($p < 0.05$) following one-way ANOVA. Bold letters highlight the highest value within each variable.
